# Behandlungszufriedenheit bei Patienten mit zervikaler Dystonie: Subgruppenanalyse von INTEREST-IN-CD-2

**DOI:** 10.1007/s00115-021-01120-1

**Published:** 2021-05-03

**Authors:** Chi Wang Ip, Axel Schramm, Pascal Maisonobe, Emma Zaragatski, Thomas Sycha

**Affiliations:** 1 Neurologische Klinik und Poliklinik, Universitätsklinikum Würzburg, Würzburg, Deutschland; 2 Neuropraxis Fürth, Fürth, Deutschland; 3 Ipsen Pharma GmbH, Boulogne-Billancourt, Frankreich; 4 Ipsen Pharma GmbH, Einsteinstr. 174, 81677 München, Deutschland; 5 Universitätsklinik für Neurologie, Medizinische Universität Wien, Wien, Österreich

**Keywords:** Botulinumtoxin, Zervikale Dystonie, Therapiezufriedenheit, INTEREST-IN-CD2-Studie, TWSTRS, Botulinum toxin, Cervical dystonia, Treatment satisfaction, INTEREST-IN-CD‑2 trial, TWSTRS

## Abstract

**Hintergrund:**

Obwohl Botulinumtoxin‑A (BoNT-A) von Leitlinien als First-line-Therapie der fokalen zervikalen Dystonie (ZD) empfohlen wird, existieren kaum Langzeitdaten zu den Behandlungsmodalitäten in der klinischen Routine.

**Fragestellung:**

Die vorliegende Subgruppenanalyse untersuchte Patientenzufriedenheit und Symptomkontrolle unter Berücksichtigung von Behandlungsmodalitäten der BoNT-A-Therapie zwischen ZD-Patienten in Deutschland und Österreich (DE/AT, *n* = 79) und der internationalen Gesamtkohorte (*n* = 995).

**Material und Methoden:**

INTEREST-IN-CD2 war eine prospektive, multizentrische, longitudinale Beobachtungsstudie, die über 3 Jahre der Therapie erwachsener Patienten mit idiopathischer ZD unter BoNT-A-Behandlung folgte. Primärer Endpunkt war die Patientenzufriedenheit mit der Therapie gemessen an der maximalen Zufriedenheit zwischen 2 Injektionen und der Zufriedenheit zum Zeitpunkt der Reinjektion.

**Ergebnisse:**

Die Therapiezufriedenheit im Wirkmaximum war in beiden Populationen im Studienverlauf stabil und vergleichbar gut (82,3–92,7 % bzw. 85,0–89,9 %). Mit nachlassender BoNT-A-Wirkung zum Ende des Behandlungsintervalls sank die Zufriedenheit ab: Zu Studienbeginn in beiden Gruppen ähnlich (54,2 % vs. 51,4 %), fiel sie numerisch in der der DE/AT-Gruppe bis auf 32,7 % ab, blieb dagegen in der Gesamtpopulation stabil. Die Toronto Western Spasmodic Torticollis Rating Scale(TWSTRS)- und Tsui-Scores zeigten keine wesentlichen Unterschiede zwischen der DE/AT-Gruppe und der Gesamtpopulation.

**Schlussfolgerungen:**

Die Studie bestätigt insgesamt eine gute klinische Symptomkontrolle durch BoNT‑A. Die im Vergleich von DE/AT zur internationalen Gesamtkohorte gesehenen numerischen Unterschiede in der aktuellen Zufriedenheit sind möglicherweise bedingt durch abweichende Anteile BoNT-A-naiver Patienten beider Gruppen, da diese unterschiedliche Zufriedenheit als vorbehandelte Patienten äußerten.

**Zusatzmaterial online:**

Die Onlineversion dieses Beitrags (10.1007/s00115-021-01120-1) enthält weitere Infomaterialien. Beitrag und Zusatzmaterial stehen Ihnen auf www.springermedizin.de zur Verfügung. Bitte geben Sie dort den Beitragstitel in die Suche ein, das Zusatzmaterial finden Sie beim Beitrag unter „Ergänzende Inhalte“.

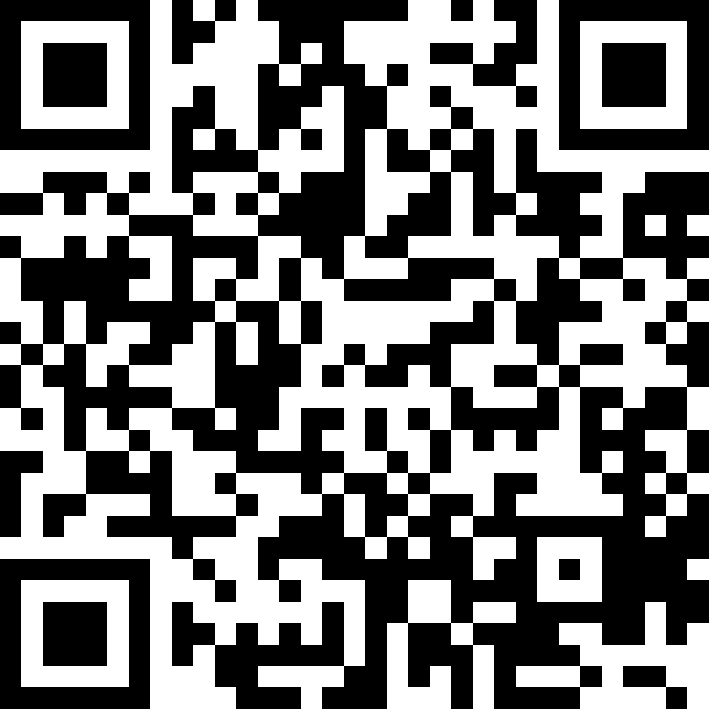

## Abstract (en)

**Background:**

Even though treatment guidelines recommend botulinum neurotoxin A (BoNT-A) as first line treatment for primary cervical dystonia (CD), there are only limited data on how BoNT-A-injections are administered in routine clinical practice.

**Objective:**

This subgroup analysis evaluated patient satisfaction and symptom control under consideration of BoNT‑A treatment modalities in German and Austrian CD patients (DE/AT, *n* = 79) compared to the full international cohort (*n* = 995).

**Material and methods:**

The INTEREST-IN-CD2 was a prospective, multicenter, longitudinal observational study. Course of treatment in adult primary CD patients under BoNT‑A treatment was assessed over a time period of 3 years. Primary outcome measure was the long-term satisfaction of patients with treatment, measured as maximum satisfaction between two consecutive injections as well as satisfaction at the time of reinjection.

**Results:**

Treatment satisfaction at the maximum effect was stable and comparably good in both populations during the study (82.3–92.7% and 85.0–89.9%). Satisfaction decreased with decreasing BoNT‑A effect at the end of the treatment interval: it was comparable at the start of the study in both groups (54.2% vs. 51.4%), decreased numerically in the DE/AT group to 32.7% but remained stable in the total population. Analysis of Toronto Western Spasmodic Torticollis Rating Scale (TWSTRS) and Tsui scores did not reveal any substantial differences between the DE/AT subgroup and total cohort.

**Conclusion:**

Overall, the study confirmed good clinical symptom control by BoNT‑A. The numerical differences in the current satisfaction seen in the comparison of DE/AT to the total cohort are possibly due to different proportions of BoNT‑A naïve patients in both groups, as they expressed different levels of satisfaction than previously treated patients.

Botulinumtoxin wird bei erwachsenen Patienten mit idiopathischer zervikaler Dystonie (ZD) als First-line-Therapie empfohlen. Die INTEREST-IN-CD2-Studie untersuchte über 3 Jahre internationale Botulinumtoxin-A(BoNT-A)-Behandlungsmodalitäten sowie patientenbezogene Endpunkte unter Real-life-Bedingungen. Die vorliegende Subgruppenanalyse vergleicht die Ergebnisse der deutsch-österreichischen Kohorte mit der internationalen Gesamtkohorte, um Einblicke in Therapiepraxis, Therapieerfolge und Therapiezufriedenheit im deutschsprachigen Raum zu erhalten.

## Hintergrund

Die ZD ist die häufigste Form der fokalen Dystonien bei Erwachsenen. Sie ist durch eine unwillkürliche tonische Neigung oder Drehung des Nackens und eine daraus resultierende Fehlstellung des Kopfes charakterisiert [4, 18]. Der negative Einfluss der ZD auf das alltägliche Leben und die Lebensqualität wurde bereits durch zahlreiche Studien belegt [4, 5, 11, 12, 18].

First-line-Therapie der ZD gemäß Leitlinienempfehlung ist die Behandlung mit BoNT [16]. Die Anwendung von BoNT‑A im klinischen Alltag, der Verlauf der Erkrankung unter BoNT-A-Therapie und die Patientenzufriedenheit sind bisher nicht hinreichend beschrieben [5, 8, 9].

Zwar sind placebokontrollierte Studien der Goldstandard für die Wirksamkeitsanalyse einer Therapie, sie können aber mit strengen Ein- und Ausschlusskriterien und limitierten Endpunkten viele Schlüsselfragen des klinischen Alltags nicht beantworten. Zudem rücken bei Kostenträgern und regulatorischen Instanzen patientenbezogene Endpunkte immer mehr in den Vordergrund. Als entscheidender Parameter für die BoNT-A-Therapie gilt die Patientenzufriedenheit, die direkt mit der Bereitschaft zu der i. d. R. über Jahrzehnte erforderlichen Behandlung korreliert [17, 20].

Die internationale Beobachtungsstudie INTEREST-IN-CD2 untersuchte die langfristige Patientenzufriedenheit bezüglich der Symptomkontrolle über insgesamt 3 Jahre [2, 3, 10]. Im Rahmen einer Subgruppenanalyse wurden nun die Studiendaten der deutsch-österreichischen Patientenkohorte (DE/AT) der internationalen Gesamtkohorte gegenübergestellt.

## Methodik

INTEREST-IN-CD2 war eine prospektive, multizentrische, longitudinale Beobachtungsstudie an 113 Studienzentren in 34 Ländern, die dem Therapieverlauf von Patienten mit idiopathischer ZD unter BoNT-Therapie folgte. Für die DE/AT-Subgruppe wurden Daten aus allen 6 deutschen und dem österreichischen Behandlungszentrum eingeschlossen. Zur Vermeidung der Ergebnisverzerrung durch ungleichmäßige Verteilung von Patientenzahlen je Studienzentrum war der Einschluss auf maximal 8 bis 12 Patienten pro Studienzentrum begrenzt. Weitere Details zu Studienpopulation, -durchführung und Datenauswertung wurden bereits an anderer Stelle publiziert [2, 10].

### Primäre und sekundäre Endpunkte

Primärer Endpunkt war die vom Patienten bewertete Zufriedenheit mit der erzielten Symptomkontrolle, definiert als a) höchste Zufriedenheit seit der letzten BoNT-A-Injektion (Frage an die Patienten: „Was war Ihr höchstes Zufriedenheitslevel zu einem beliebigen Zeitpunkt seit der letzten Visite im Hinblick auf die Kontrolle Ihrer ZD-assoziierten Symptome?“) sowie b) tagesaktuelle Zufriedenheit mit der Symptomkontrolle am Ende des Injektionsintervalls (Frage an die Patienten: „Wie zufrieden sind Sie heute im Hinblick auf die Kontrolle Ihrer ZD-assoziierten Symptome?“). Die Zufriedenheit wurde auf einer 5‑Punkte-Likert-Skala angegeben (1: vollkommen zufrieden, 2: relativ zufrieden, 3: weder zufrieden noch unzufrieden, 4: relativ unzufrieden, 5: vollkommen unzufrieden). Werte von 1 bis 2 wurden als Zufriedenheit gewertet, Werte von 3 bis 5 als Unzufriedenheit. Sekundäre Endpunkte waren wie folgt: demografische Patientendaten zu Studienbeginn, Toronto Western Spasmodic Torticollis Rating Scale (TWSTRS-)Score, Tsui-Subscore D (Schwere und Dauer des Tremors), Dystoniemuster, Injektionsparameter (Dosis und Volumen, injizierte Muskeln, Anzahl der Injektionsstellen, Nutzung einer Injektionshilfe), anatomische Lage und Veränderung der Fehlstellung, Erkrankungshistorie. Das zu Studienbeginn angewandte Reinjektionsschema wurde dokumentiert (i.e. festgelegte, flexible oder gemischte Intervalle). Die Erhebung von Daten zu unerwünschten Ereignissen (UE) gehörte nicht zu den Endpunkten der Studie. Gemäß den europäischen Leitlinien zur Arzneimittelsicherheit wurden die Studienzentren aufgefordert, alle schwerwiegenden UE und alle im Zusammenhang mit der Therapie stehenden UE an den Studiensponsor zu melden.

## Ergebnisse

### Studienpopulation und Baseline-Charakteristika

**Figure 1:**
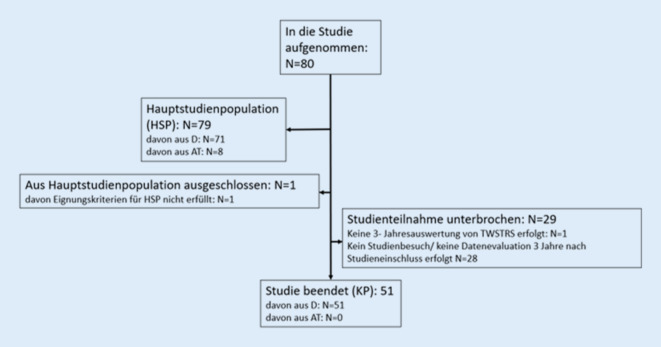

Der Patientenfluss der DE/AT-Kohorte ist in Abb. 1 dargestellt. Es wurden 79 Patienten in die Hauptstudienpopulation (HSP) aufgenommen. Die Baseline-Charakteristika der DE/AT- sowie der internationalen Gesamtkohorte sind in Tab. 1 zusammengefasst. Relevante Unterschiede zeigten sich u. a. bei der Verwendung von Injektionshilfen für mindestens einen Muskel (DE/AT: 45,6 %, Gesamtkohorte: 35,4 %), dabei insbesondere bei der Sonographie (13,9 % vs. 3,4 %). Die zur Baseline und im Studienverlauf am häufigsten injizierten Muskeln waren in beiden Gruppen identisch. Größere Unterschiede zeigten sich bei Injektionen in den M. trapezius (51,9 % vs. 64,3 %) sowie in der Mm.-obliqui-capitis-Gruppe (22,8 % vs. 5,4 %, je DE/AT vs. Gesamtkohorte). Der Anteil BoNT-A-naiver Patienten war in DE/AT deutlich höher als in der globalen Kohorte (25 % vs. 9 %).

**Table 1:** 

Demographie	DE/AT (*n* = 79)	Gesamt (*n* = 995)
*Alter (Jahre); Mittel* *±* *SA*	55,5 ± 12,3	54,8 ± 13,1
*Weiblich/männlich (n [%])*	50 (63,3)/29 (36,7)	677 (68,0)/318 (32,0)
*Anteil BoNT-A-naiver Patienten (n [%])*	20 (25,0)	111 (9,0)
*Zeit seit Diagnose (Jahre), Mittel* *±* *SA*	10,0 ± 9,2	8,7 ± 8,1
*TWSTRS-Score, Mittel* *±* *SA*		
Gesamt	27,19 ± 10,69	31,59 ± 13,04
Schweregrad	13,62 ± 4,66	15,90 ± 5,66
Behinderung	7,97 ± 5,76	9,39 ± 6,28
Schmerz	5,59 ± 4,20	6,29 ± 4,86
*Dominierendes ZD-Muster (n [%])*		
Rotation	47 (59,5)	657 (66,2)
Laterokollis	21 (26,6)	228 (23,0)
Retrokollis	5 (6,3)	59 (5,9)
Antekollis	3 (3,8)	20 (2,0)
Lateraler Shift	2 (2,5)	15 (1,5)
Sagittaler Shift	0 (0)	10 (1,0)
Nicht anwendbar	1 (1,3)	4 (0,4)
*Verwendung einer Injektionshilfe für ≥* *1 Muskel (%)*	45,6	35,4
EMG für ≥ 1 Muskel (%)	30,4	32,1
Elektrostimulation für ≥ 1 Muskel (%)	0	0,3
Sonographie für ≥ 1 Muskel (%)	13,9	3,4
CT für ≥ 1 Muskel (%)	1,3	0,1
*Häufigste injizierte Muskeln (%)*		
M. splenius capitis	93,7	87,3
M. sternocleidomastoideus	88,6	82,6
M. trapezius	51,9	64,3
M. levator scapulae	46,8	40,9
M. semispinalis capitis	34,2	26,9
Mm. obliqui capitis	22,8	5,4

*BoNT‑A* Botulinumtoxin‑A, *CT* Computertomographie, *EMG* Elektromyographie, *SA* Standardabweichung, *TWSTRS* Toronto Western Spasmodic Torticollis Rating Scale, *ZD* zervikalen Dystonie

### BoNT-A-Therapie

Die Behandlungsparameter beider Kohorten sind in Tab. 2 zusammengefasst. Alle Patienten in DE/AT erhielten ein BoNT-A-Produkt. Abobotulinumtoxin‑A (AboBoNT‑A) und Incobotulinumtoxin‑A (IncoBoNT‑A) wurden in DE/AT höher dosiert als in der Gesamtpopulation. Die Injektionsintervalle waren in DE/AT im Median kürzer (97,82 vs. 107 Tage), die Anzahl der injizierten Muskeln etwas höher als global (Median: 5,0 vs. 4,25). Auf die Frage, ob in den DE/AT-Studienzentren feste oder flexible Injektionsintervalle oder eine Mischung aus beidem praktiziert würde, antwortete ein Studienzentrum mit „flexible Intervalle“, drei mit „Mix aus beidem“ während drei Zentren sich nicht äußerten.

**Table 2:** 

Injektionsparameter	DE/AT (*n* = 79)	Gesamt (*n* = 995)
*Anzahl Injektionszyklen, Median (Range)*	11 (1–12)	10 (1–17)
*Dosis (Units), Median (Range)*		
AboBoNT‑A (*n* = 49 vs. 614)	600 (275–1186,4)	500,0 (50,0–1833,3)
IncoBoNT‑A (*n* = 6 vs. 44)	260 (156,7–514,3)	198,6 (45,6–514,3)
OnaBoNT‑A (*n* = 16 vs. 186)	153,75 (80–263,3)	150,0 (13,3–500,0)
*Anzahl injizierte Muskeln, Median (Range)*	5,00 (2,0–10,1)	4,25 (1,0–18,2)
*Injektionsintervall (Tage)*		
Mittel ± SA	101,69 ± 15,76	121,5 ± 48,4
Median (Range)	97,82 (57,0–184,7)	107,0 (44,0–547,0)
*Mittlere Injektionsintervallkategorie (n [%])*		
< 12 Wochen	1 (1,3)	11 (1,1)
12–16 Wochen	67 (84,8)	580 (58,6)
> 16 Wochen	11 (13,9)	399 (40,3)

*AboBoNT‑A* AbobotulinumtoxinA, *IncoBoNT‑A* IncobotulinumtoxinA, *OnaBoNT‑A* OnabotulinumtoxinA, *SA* Standardabweichung

### Symptomkontrolle mit BoNT-A (klinisches Ansprechen)

**Figure 2:**
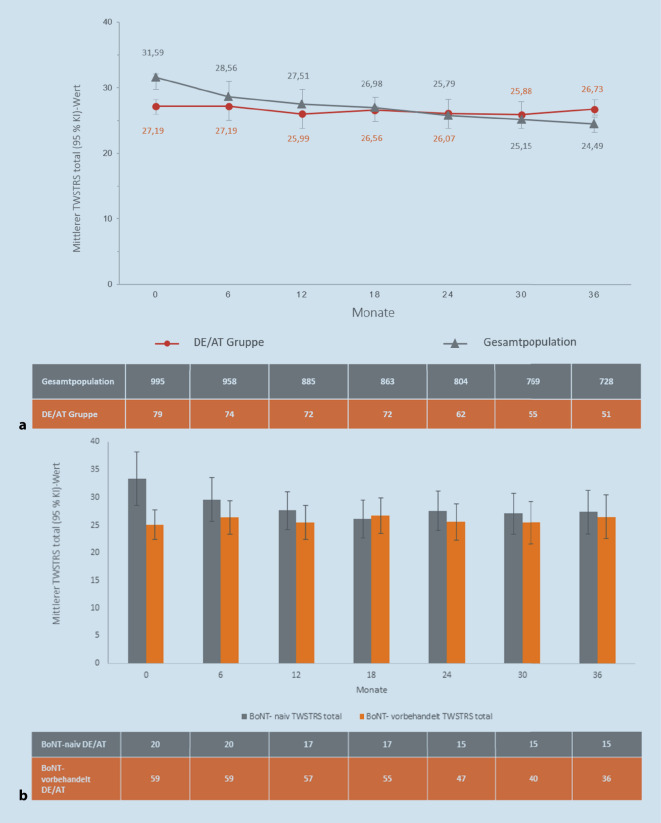

Der mittlere TWSTRS-Gesamtscore sank global im Studienverlauf weitgehend konstant von 31,59 auf 24,49 (mittlere Reduktion: −6,97 ± 11,56 Punkte). Demgegenüber lag der TWSTRS-Gesamtscore der DE/AT-Population mit 27,19 bereits zur Baseline niedriger und blieb im Verlauf weitgehend ähnlich (Abb. 2a). Bei BoNT-A-naiven Patienten in DE/AT lag der TWSTRS gesamt bei 33,35 ± 10,25 zu Studienbeginn und konnte nach 36 Monaten auf 27,27 ± 7,11 reduziert werden, während er sich bei BoNT-A-vorbehandelten Patienten im Studienverlauf kaum änderte (Abb. 2b).

**Figure 3:**
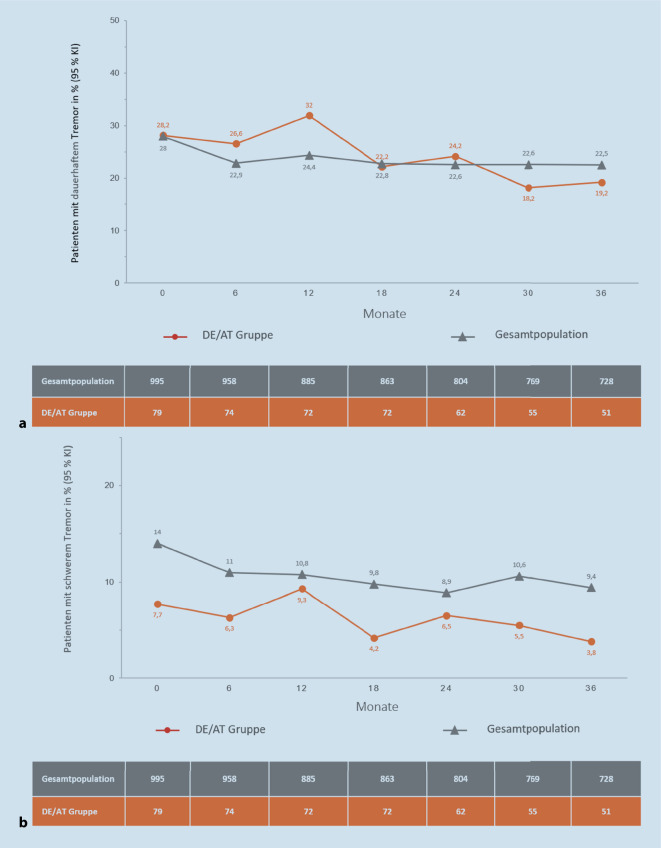

Der Anteil von Patienten mit ZD-assoziiertem Tremor, dargestellt in der Tsui-Kategorie „Tsui Tremor-Dauer“, verbesserte sich im Studienverlauf in beiden Populationen leicht (Abb. 3a). Ähnliches zeigte sich für den Patientenanteil mit schwerem Tremor (Tsui-Kategorie „Tsui Tremor-Schweregrad“). Dieser war in der Gesamtpopulation höher als in DE/AT (Baseline: 14 % vs. 7,7 %) und fiel in beiden Kohorten im Behandlungsverlauf ab (Abb. 3b).

### Subjektive Zufriedenheit mit der Symptomkontrolle

**Figure 4:**
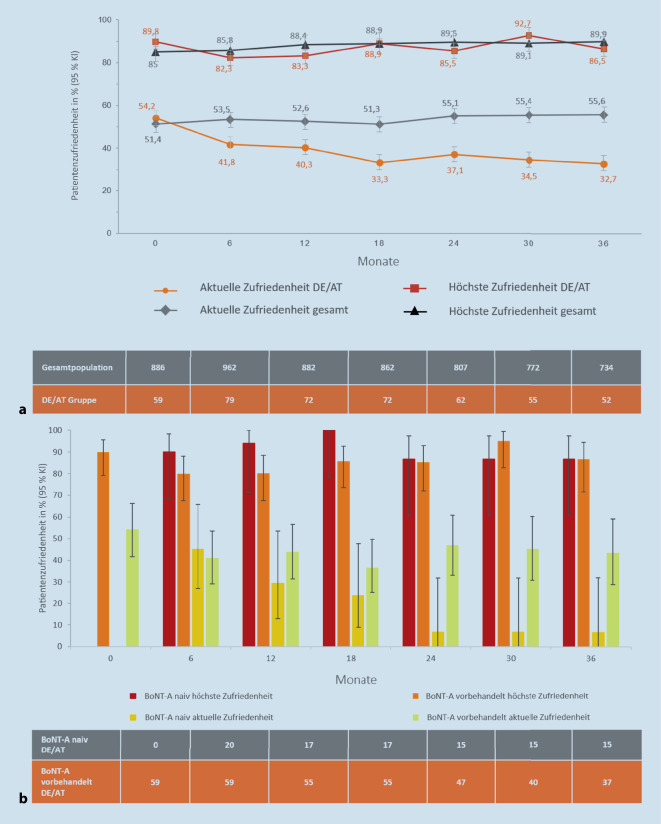

Die höchste Therapiezufriedenheit war in beiden Populationen vergleichbar und im Studienverlauf weitgehend konstant (Baseline: 89,8 % DE/AT vs. 85,0 % Gesamtpopulation; Abb. 4a). Im Vergleich der höchsten Zufriedenheit der DE/AT-BoNT-A-naiven mit -BoNT-A-vorbehandelten Patienten waren die naiven in den ersten 24 Monaten zufriedener als die vorbehandelten Patienten, wobei sich die höchsten Zufriedenheiten im Studienverlauf anglichen (Abb. 4b).

Die „aktuelle Zufriedenheit“ am Ende jedes Injektionsintervalls war in DE/AT sowie in der Gesamtgruppe deutlich niedriger als die höchste Zufriedenheit (Baseline: DE/AT 54,2 %, Gesamtpopulation 51,4 %) und fiel in der DE/AT-Population über 36 Monate numerisch, aber kontinuierlich auf bis zu 32,7 % ab (Abb. 4a). Dem zugrunde liegend war hier eine sinkende aktuelle Zufriedenheit der BoNT-A-naiven Teilgruppe der DE/AT-Patienten (Abb. 4b).

DE/AT-Patienten wählten Punkt 3 auf der Likert-Skala für die Beschreibung ihrer Therapiezufriedenheit häufiger als Patienten der internationalen Gesamtkohorte (30,6 % vs. 20,8 % nach 36 Monaten; +62 % für DE/AT; [2], siehe elektronisches Zusatzmaterial).

### Sicherheit

Wie für die globale Studiengruppe berichtet, traten auch in der DE/AT-Subgruppe keine neuen und unerwarteten Nebenwirkungen unter BoNT‑A auf. Die berichteten Nebenwirkungen entsprachen denjenigen, welche bei der Verwendung von BoNT‑A bei ZD bereits bekannt sind.

## Diskussion

Die INTEREST-IN-CD2-Studie ist die derzeit größte prospektiv-longitudinale Kohortenstudie, die BoNT-behandelte ZD-Patienten über einen Zeitraum von 3 Jahren folgte [2, 3, 10]. Den Schwerpunkt der Analyse bildete die Therapiezufriedenheit, die wegen des Zusammenhangs mit der Therapieadhärenz immer mehr in den Fokus klinischer Studien rückt [1, 4]. In der hier beschriebenen Subgruppenanalyse lassen die geringen Dropout-Raten sowohl in der internationalen Gesamtkohorte (ca. 24 %) als auch in der DE/AT-Population (ca. 36 %) auf eine generell hohe Therapieadhärenz schließen.

Das Collum-Caput(Col-Cap)-Konzept wurde in Deutschland [13] entwickelt und findet hier häufige Anwendung. Seine Beachtung beinhaltet eine differenzierte Diagnose und Injektion unter Anwendung bildgebender Verfahren (z. B. Ultraschall) und resultiert in der Injektion schwer erreichbarer Muskeln wie dem M. obliquus capitis inferior [13, 14]. In der DE/AT-Gruppe wurde die Obliquus-capitis-Gruppe häufiger injiziert als in der globalen Gruppe und es fanden häufiger ultraschallgestützte Injektionen statt, was eine stärkere Anwendung des Col-Cap-Konzeptes in dieser Kohorte bestätigt.

Klinisch profitierten DE/AT-Patienten vor allem von einer Verbesserung des Tsui-Tremor-Subscores sowohl bei der Dauer als auch bei der Schwere des Tremors. Die BoNT-A-Behandlung reduzierte ZD-assoziierte Symptome bei naiven Patienten, gemessen an TWSTRS total, bereits zum ersten Beobachtungszeitpunkt nach Therapiebeginn. Zu betonen ist, dass TWSTRS- und Tsui-Werte am Ende der Injektionszyklen bei schwindendem BoNT-A-Effekt ermittelt wurden und daher nicht das gesamte Maß der Symptomreduktion bei maximaler BoNT-A-Wirkung zeigen [1, 15]. In beiden Populationen wurden keine Veränderung des Dystoniemusters im Therapieverlauf beobachtet.

Die maximale Therapiezufriedenheit war in beiden Kohorten mit knapp 90 % über den Studienverlauf hinweg sehr hoch. Die „aktuelle Zufriedenheit“ zum Ende jedes Therapiezyklus lag in beiden Kohorten deutlich niedriger, die nachlassende Wirkung der BoNT-A-Injektionen am Ende der Intervalle lässt dies auch erwarten [1, 15, 19]. Es ist bekannt, dass die zum Zyklusende wiederkehrenden Symptome sich auf die Lebensqualität und das emotionale Wohlbefinden von ZD-Patienten auswirken [5, 12]. Während in der globalen Gruppe die „aktuelle Zufriedenheit“ im Zeitverlauf konstant blieb, fiel sie in der DE/AT-Gruppe numerisch ab. Insgesamt war der prozentuale Anteil BoNT-A-naiver Patienten in der DE/AT-Gruppe im Beobachtungszeitraum mindestens doppelt so hoch wie in der globalen Kohorte. Da diese Patienten eine besonders in der zweiten Studienhälfte geringere Therapiezufriedenheit äußerten als therapieerfahrenere, vorbehandelte Patienten, trägt dies möglicherweise zum numerischen Rückgang der aktuellen Zufriedenheit in DE/AT bei. Aus klinischer Sicht ergeben sich mit stabilen/verbesserten Befunden auch in der BoNT-A-naiven Gruppe keine objektivierbaren Hinweise für eine Verschlechterung der Symptomkontrolle bezogen auf „aktuelle Zufriedenheit“, z. B. anhand von TWSTRS.

Abweichende Therapiezufriedenheiten zwischen BoNT-A-naiven und -vorbehandelten Patienten wurden bereits beschrieben [3]. Eine mögliche Erklärung wäre, dass BoNT-A-naiven Patienten eine objektive Einschätzung des Therapieerfolges schwerfallen kann, auch eine Gewöhnung an die therapiebedingte Symptomverbesserung ist bekannt. Dies wurde zur BoNT-A-Behandlung der Dystonie bereits unter dem Begriff „Flitterwochen-Effekt“ publiziert [6]. Auch bei konstanter Symptomkontrolle wird der Therapieerfolg nach einiger Zeit nicht mehr als solcher wahrgenommen, die Zufriedenheit sinkt [6]. Erst jahrelanger Umgang mit der Erkrankung und Erfahrungen mit den Höhen und Tiefen des Therapieansprechens können den Betroffenen wieder einen objektiveren Blick auf die erzielte Symptomkontrolle ermöglichen.

Auch gesundheitssystemassoziierte Besonderheiten in der Arzt-Patienten-Kommunikation könnten zu einer geringeren Zufriedenheit der DE/AT-Patienten beitragen. Beim Arzt-Patienten-Gespräch in Deutschland herrscht ein höherer Zeitdruck als in vielen europäischen Ländern oder den USA [7]. Dieser könnte es dem Arzt erschweren, die Behandlungsziele ausführlich zu kommunizieren und eine realistische Erwartungshaltung an das Therapieansprechen zu erzeugen. Eine hohe Erwartungshaltung, insbesondere hinsichtlich der Schmerzfreiheit und einer reduzierten Muskelanspannung, wurde bei ZD-Patienten bereits dokumentiert [4]. Mehr als die Hälfte der Betroffenen erwartete, unter der Therapie wieder zur normalen täglichen Routine zurückkehren zu können [4]. Diese Erwartungshaltung könnte zu einer besonders kritischen Einstellung gegenüber der Therapie, insbesondere bei BoNT-A-naiven, also unerfahrenen Patienten führen.

Limitationen der Subgruppenanalyse liegen vor allem in der begrenzten Patientenzahl innerhalb der DE/AT-Kohorte (79 vs. 995 in der globalen Kohorte), der geringen Zahl von Patienten, die die Studie in DE/AT beendet haben sowie der rein deskriptiven Auswertung der Daten. Zu beachten ist weiterhin die geringe Anzahl BoNT-A-naiver Patienten in DE/AT. Zwar zählt der im Gegensatz zu vielen anderen Studien stehende Einschluss aller in DE/AT zugelassenen BoNT-A-Produkte grundsätzlich zu den Stärken der INTEREST-IN-CD2-Studie, allerdings lassen die geringen Patientenzahlen in der DE/AT-Kohorte keine differenzierten Aussagen für die verschiedenen Präparate zu. Weitere Einschränkungen ergeben sich aus der Messung klinisch relevanter Parameter wie TWSTRS und Tsui am Ende der Injektionszyklen, also bei nachlassender Wirksamkeit der verabreichten Präparate.

## Fazit für die Praxis

Die subjektive Patientenzufriedenheit von Patienten mit zervikaler Dystonie (ZD), die mit Botulinumtoxin‑A (BoNT‑A) behandelt werden, hängt nicht nur von der objektiven Wirksamkeit und dem ärztlich festgestellten Behandlungserfolg der BoNT-A-Therapie ab. Eine klare und patientenzentrierte ärztliche Hinleitung zu einer realistischen Patientenerwartungshaltung spielt möglicherweise eine wichtige Rolle in der Wahrnehmung von Therapieerfolgen und sollten bei der Behandlung der Patienten mit in Betracht gezogen werden.

## Supplementary Information





## References

[1] Bensmail D (2014). Satisfaction with botulinum toxin treatment in post-stroke spasticity: results from two cross-sectional surveys (patients and physicians). J Med Econ.

[2] Colosimo C, INTEREST IN CD2 study group, study group (2019). How satisfied are cervical dystonia patients after 3 years of botulinum toxin type A treatment? Results from a prospective, long-term observational study. J Neurol.

[3] Colosimo C (2020). Cumulative effects of long-term treatment with abobotulinumtoxinA in cervical dystonia: findings from a prospective, observational study. J Neurol Sci.

[4] Comella C, Bhatia K (2015). An international survey of patients with cervical dystonia. J Neurol.

[5] Comella C (2020). Patient perspectives on the therapeutic profile of botulinum neurotoxin type A in cervical dystonia.. J Neurol.

[6] Hallett M, Poewe W (2008). Therapeutics of Parkinson’s disease and other movement disorders.

[7] Irving G (2017). International variations in primary care physician consultation time: a systematic review of 67 countries. BMJ Open.

[8] Jochim A (2019). Treatment of cervical dystonia with abo-and onabotulinumtoxinA: long-term safety and efficacy in daily clinical practice. J Neurol.

[9] Jost WH (2019). Effectiveness of botulinum neurotoxin type A injections in naïve and previously-treated patients suffering from Torti-or Laterocollis or-caput: results from a German-Austrian open-label prospective post-marketing surveillance study. J. Neurol. Sci..

[10] Misra VP, INTEREST IN CD2 study group, study group (2018). INTEREST IN CD2, a global patient-centred study of long-term cervical dystonia treatment with botulinum toxin. J Neurol.

[11] Müller J (2002). The impact of blepharospasm and cervical dystonia on health-related quality of life and depression. J Neurol.

[12] Poliziani M (2016). Striving for more good days: patient perspectives on botulinum toxin for the treatment of cervical dystonia. PPA.

[13] Reichel G (2011). Cervical dystonia: a new phenomenological classification for botulinum toxin therapy. Bas Gang.

[14] Schramm A (2015). Relevance of sonography for botulinum toxin treatment of cervical dystonia: an expert statement. J. Neural. Transm..

[15] Sethi KD (2012). Satisfaction with botulinum toxin treatment: a cross-sectional survey of patients with cervical dystonia. J Med Econ.

[16] Simpson DM (2016). Practice guideline update summary: Botulinum neurotoxin for the treatment of blepharospasm, cervical dystonia, adult spasticity, and headache: Report of the Guideline Development Subcommittee of the American Academy of Neurology. Neurol.

[17] Skogseid IM, Kerty E (2005). The course of cervical dystonia and patient satisfaction with long-term botulinum toxin A treatment. Eur J Neurol.

[18] Stacy M (2008). Epidemiology, clinical presentation, and diagnosis of cervical dystonia. Neurol Clin.

[19] Truong D, Global Dysport Cervical Dystonia Study Group, Global Dysport Cervical Dystonia Study Group (2010). Long-term efficacy and safety of botulinum toxin type A (Dysport) in cervical dystonia. Park Rel Dis.

[20] Weaver M (1997). Issues in the measurement of satisfaction with treatment. Am J Man Care.

