# Supplementary material for: Treatment satisfaction in patients with cervical dystonia: Subgroup analysis of INTEREST-IN-CD-2
Source: Nervenarzt. 2021 May 3;92(12):1268–75. [Article in German] doi: 10.1007/s00115-021-01120-1 (PMC8648643; doi:10.1007/s00115-021-01120-1)
Supplement: Supplementary file 1 [file 115_2021_1120_MOESM1_ESM.pdf]

Anhang (Supplement)

Tabelle 1: Verwendung des Punktes 3 auf der Likert Skala (weder zufrieden noch unzufrieden):

Heutige Zufriedenheit DE/AT vs. global (Hauptstudienpopulation), 95% KI

Monate	DE/AT	global
0	**22,0** (13,2- 34,3)	**15,2** (13,0-17,8)
6	**26,6** (18,0-37,3)	**16,7** (14,5-19,2)
12	**30,6** (21,1-42,0)	**18,8** (16,4-21,5)
18	**30,6** (21,1-42,0)	**20,8** (18,2-23,6)
24	**32,3** (21,9-44,7)	**19,0** (16,4- 21,8)
30	**36,4** (24,9-49,6)	**16,8** (14,4-19,6)
36	**34,6** (23,1-48,2)	**18,4** (15,8-21,4)
